# Ag-lignin hybrid nanoparticles for high-performance solar absorption in photothermal antibacterial chitosan films

**DOI:** 10.1016/j.isci.2023.108058

**Published:** 2023-09-25

**Authors:** Jinrong Liu, Mika H. Sipponen

**Affiliations:** 1 Department of Materials and Environmental Chemistry, Stockholm University, 106 91 Stockholm, Sweden; 2 Wallenberg Wood Science Center, Department of Materials and Environmental Chemistry, Stockholm University, 106 91 Stockholm, Sweden

**Keywords:** Biotechnology, Biomass, Materials science, Materials chemistry

## Abstract

There is an urgent need for antimicrobial films based on sustainable resources and production methods. In this study, we present a bio-based nanocomposite film composed of chitosan (∼60 wt %), lignin nanoparticles (LNPs, ∼40 wt %), a small amount of glutaraldehyde (1.5 wt %), and a trace level of silver nanoparticles (AgNPs, 0.072 wt %). The uniform dispersion with LNPs prevented aggregation of metallic silver, resulting in small (diameter 3.3 nm) AgNPs. The nanocomposite film absorbs 89% of radiation across the entire solar spectrum and exhibits a remarkable photothermally triggered antibacterial effect, which is further enhanced by the dark color of lignin. Under simulated solar light illumination, the nanocomposite films demonstrated a significant reduction in viable *Escherichia coli* count compared to control scenarios. The potential applications of these nanocomposites extend to sunlight-activated antimicrobial films and coatings, addressing the growing demand for sustainable and effective antimicrobial materials.

## Highlights

• Chitosan films with increased wet mechanical strength and photothermal performance
• Lignin nanoparticles act as reducing agents to form small silver nanoparticles
• Photothermal antibacterial effect with low loading of silver-lignin hybrid NPs

## Graphical abstract

**Figure:**
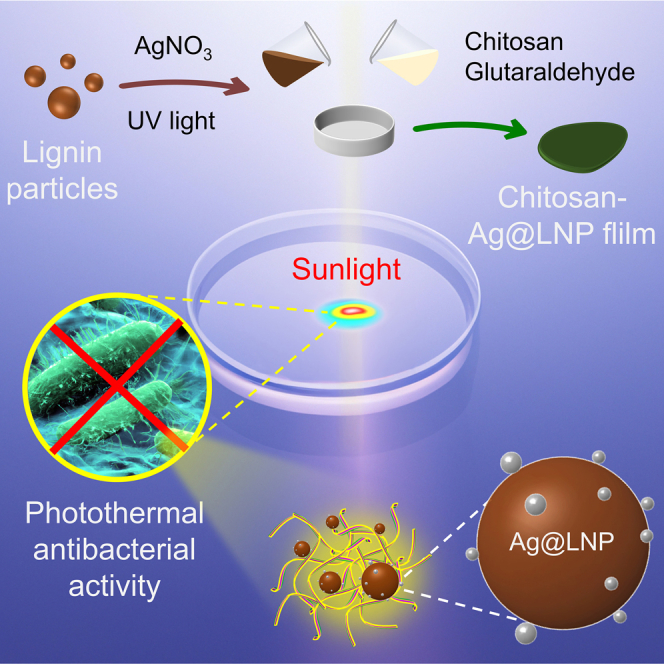

## Introduction

Attachment and proliferation of bacteria on surfaces form biofilms that are responsible for ubiquitous and persistent fouling of medical devices, biomedical implants, and processing equipment.^1^^,^^2^ The fouling process starts with protein adsorption on the surface, and is followed by the adsorption, attachment, and growth of bacteria.^3^ To cope with this problem, silver nanoparticles (AgNPs) have been studied as broad-spectrum antimicrobial agents.^4^^,^^5^^,^^6^^,^^7^^,^^8^^,^^9^^,^^10^^,^^11^^,^^12^ Recent research shows that small AgNPs (∼1–10 nm) have stronger antibacterial activity than larger particles. This is due to their ability to easily penetrate cell membranes and release high levels of silver ions.^13^^,^^14^^,^^15^^,^^16^^,^^17^^,^^18^^,^^19^ Furthermore, the surface plasmon resonance (SPR) effect of AgNPs makes them attractive as light-responsive materials for applications in diagnostics and therapeutics.^4^ Such antimicrobial photothermal therapy is promising especially when considering the use of affordable light energy, for example, sunlight for photoactivated sterilization.^20^

Considering the large scale of the aforementioned challenge, there is a need for sustainable bio-based antibacterial materials such as those based on chitosan^21^^,^^22^ and cellulose^23^^,^^24^ films. Despite their merits neither chitosan nor cellulose displays inherent photocatalytic or photothermal properties, thus requiring long duration (2−72 h) to achieve sterilization. Recently, photothermal materials based on lignin have gained attention.^25^^,^^26^^,^^27^^,^^28^^,^^29^ Sourced from renewable lignocellulosic biomass, lignin is the most abundant aromatic substance available in scale from the pulp and paper and biorefinery industries. The photothermal properties of lignin arise from its conjugated aliphatic and aromatic structures as well as π-π stacking.^25^^,^^29^ Zhang et al. demonstrated photothermal-mediated antibacterial activity of lignin-AgNPs dispersion in free form and entrapped in bacterial cellulose and polyurethane films.^30^^,^^31^

Lignin is considered a multifunctional reducing and stabilizing agent to form stabilized AgNPs, because of its redox-active phenolic hydroxyl groups.^5^^,^^32^^,^^33^ Different methods have been developed to accelerate the reduction of Ag^+^ to metallic silver, such as solar light,^33^ ultraviolet (UV) light,^30^^,^^31^ heat,^34^ and microwave irradiation.^35^^,^^36^ Compared to crude technical lignins, lignin nanoparticles (LNPs) are attractive reducing agents because of their high-surface energy and colloidal stability, preventing the aggregation of metal nanoparticles. The pH-instability of LNP is a major challenge when using conventional methods which require alkaline conditions for the synthesis of AgNPs. As a workaround, lignin has been modified through phenolation,^37^ enzymatic treatment,^5^ and using hydroxymethylated lignin for hydrothermally curable LNPs.^38^ However, the particle size of AgNPs achieved through these methods is rather large (>10 nm) which is less desirable in the antibacterial application. Additionally, the literature is lacking examples of fully bio-based photothermally activated antimicrobial nanocomposite films that possess sufficient mechanical strength and stability in aqueous media.

In the present work, we focus on addressing the above two challenges (1) preparing small AgNP (∼1 − 10 nm) using LNPs as a reducing agent under mild conditions aided by UV light; (2) developing water-resistant bio-based antibacterial films with efficient photothermal activation. The prepared films were characterized by various techniques and tested for their photothermally triggered antibacterial effect, revealing synergies between LNPs and AgNPs when used in films against a non-pathogenic strain of *Escherichia coli*.

## Results

**Figure 1:**
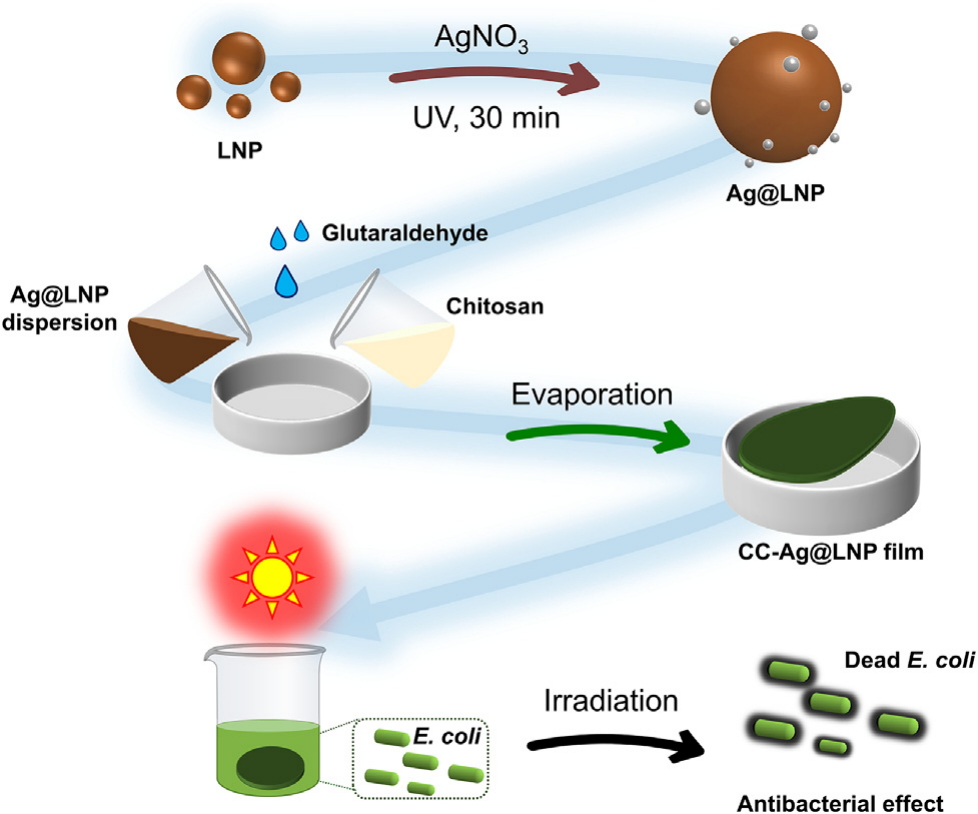
Preparation of Ag@LNP and CC-Ag@LNP film and demonstration of phtothermal antibacterial application

Our approach to prepare composited antibacterial film and demonstration of the photothermal antibacterial application is conveyed in Figure 1. Firstly, the prepared LNPs were used as a reducing agent and stabilizer to reduce Ag^+^ ions to AgNPs with the assistance of UV light, and the product was named Ag@LNP. Then the dispersion of Ag@LNP was composited with chitosan in the presence of glutaraldehyde as a cross-linker and casted into films. Chitosan was chosen as a polymer matrix because of its biodegradability^39^ and electrostatic attraction with lignin.^29^^,^^40^^,^^41^

**Figure 2:**
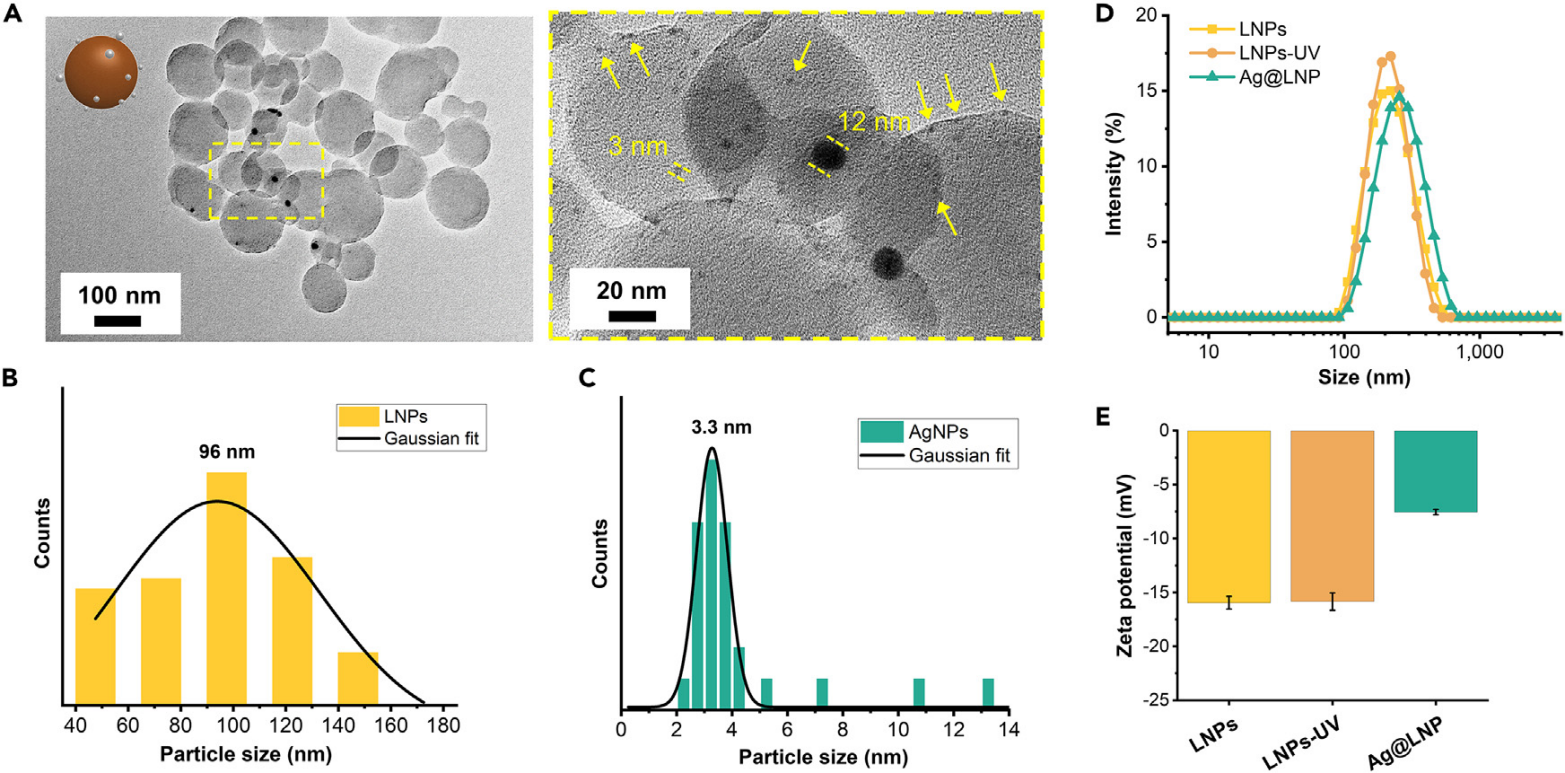
Morphology of Ag@LNP and stability of the dispersion (A) Representative TEM images of Ag@LNP. (B) Diameter distribution of LNPs measured from TEM images, including that shown in (A). A total of 64 particles were measured. (C) Diameter distribution of AgNP measured from TEM images, including that shown in (A). A total of 27 particles were measured. (D) Diameter distribution of LNPs, LNPs-UV, and Ag@LNPs from DLS measurement. (E) Zeta (ζ) potential of the aqueous dispersion of LNPs (pH 3.8), LNPs-UV (pH 3.8), and Ag@LNP (pH 3.6). Error bars represent standard deviation based on the entire population (n = 3). See also Figure S1 and Table S1.

From the TEM images of the prepared Ag@LNP, we observed spherical LNPs surrounded by AgNPs, while AgNPs appeared to be covered by a thin layer of lignin (Figure 2A). By measuring the particle size from the TEM images and fitting with a Gaussian distribution, we found that the mean size of LNPs was 96 nm and that of AgNPs was 3.3 nm (Figures 2B and 2C). The size of AgNP was within the domain that is desired for better antibacterial performance.^6^^,^^42^ Such small silver nanoparticles (<10 nm) readily release ions from their oxidized surfaces that dominate their antibacterial performance in contrast to larger particles (>10 nm) that release ions slowly.^7^^,^^13^ Therefore, the size of the silver nanoparticles in the prepared Ag@LNP is suitable for antibacterial application. LNPs demonstrate the ability to produce small AgNPs and prevent their aggregation, which is central to achieving high-quality nanocomposite films through the dispersion casting method. Table S1 summarizes different methods for forming AgNP. Among them, the UV method that we used does not require alkaline pH or heating conditions and preserves the morphology of LNP.

The interaction of LNPs and silver ions under UV irradiation can be revealed by monitoring changes in size distribution and zeta potential of the colloidal system. Dynamic light scattering showed that UV irradiation had no significant effect on the size distribution of LNPs that had a Z-average hydrodynamic diameter of 210 nm (Figure 2D). In addition, electrophoretic mobility results implied that the hydrodynamic shear surface of LNPs did not change significantly after 30 min of UV irradiation, with the zeta potential remaining at −17 mV (Figure 2E). In contrast, when silver nitrate was added to a dispersion of LNPs and then subjected to 30-min UV light irradiation, Ag@LNPs with an increased particle size of 250 nm were obtained. In addition, the zeta potential of Ag@LNP increased to −8 mV, indicating that uncharged silver particles were bound to the surface layer of LNPs. The increased zeta potential explains why Ag@LNP tended to agglomerate over extended storage durations, as shown in Figure S1, with mean particle size increasing from 257 nm to 540 nm after two weeks.

**Figure 3:**
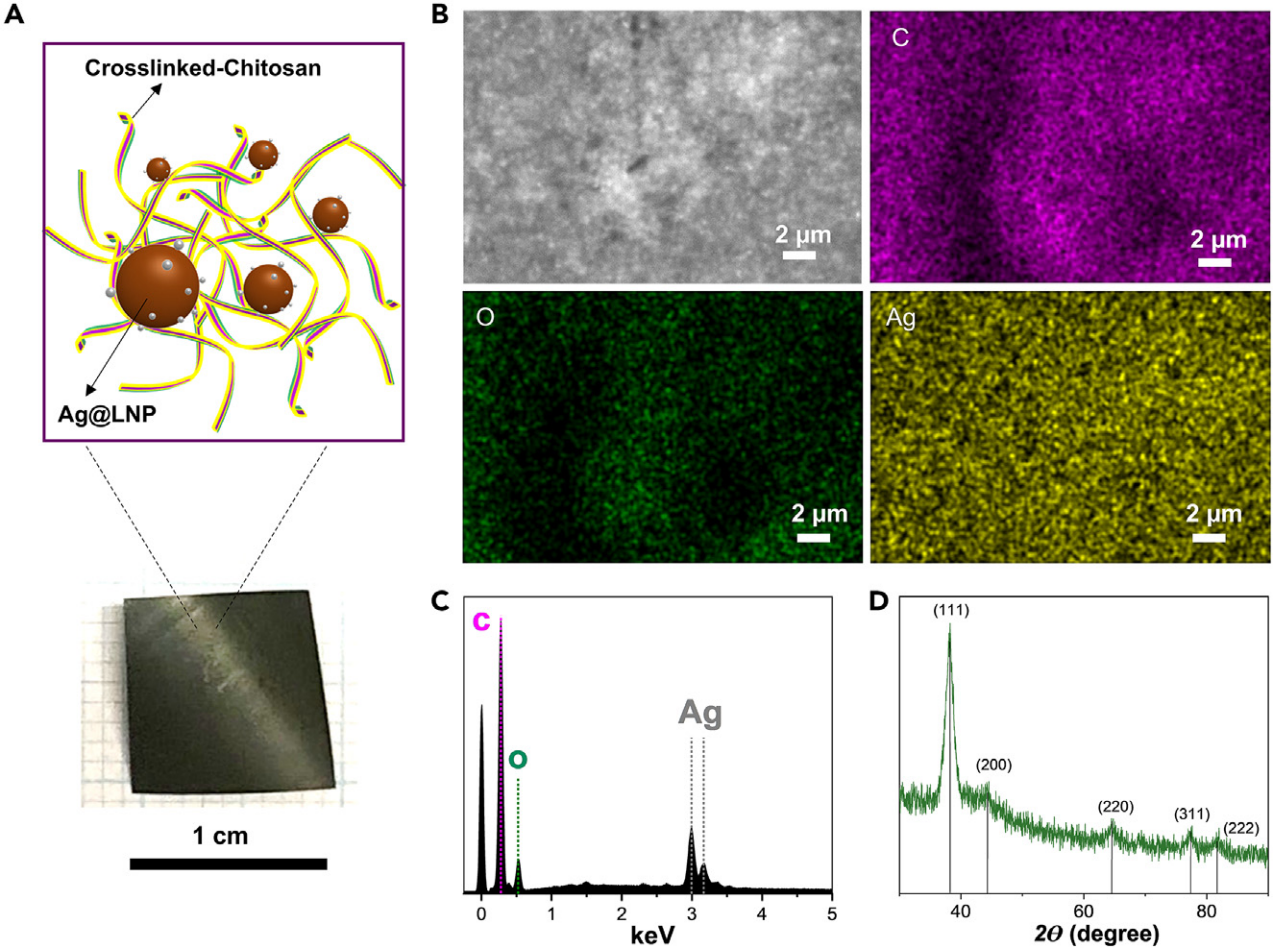
Characterization of silver in the nanocomposite films (A) Schematic illustration and digital photo of CC-Ag@LNP film. (B) SEM-EDS image of CC-Ag@LNP film and elemental mapping of carbon, oxygen, and silver. (C) EDS spectrum of the SEM image. (D) X-ray diffraction (XRD) pattern of CC-Ag@LNP film.

To overcome the long-term colloidal instability of AgNPs and address the challenge of retrieving silver nanoparticles from solid-waste incineration plants after their intended use,^32^ we opted to immobilize the AgNPs within nanocomposite films. The Ag@LNP dispersion was used to prepare a composited cross-linked chitosan-Ag@LNP (CC-Ag@LNP) film that was dark green (Figure 3A) owing to the presence of AgNPs, as revealed by SEM-EDS images and elemental mapping spectra. Silver, as a heavy element, appeared bright in the SEM image of CC-Ag@LNP compared to lighter elements such as carbon or oxygen. Elemental mapping showed that silver was uniformly distributed on the surface of the film (Figures 3B and 3C). The presence of silver in CC-Ag@LNP can be further evidenced by X-ray diffraction (XRD) pattern (Figure 3D). Peaks (*2ϴ*) at 38.2°, 44.5°, 64.7°, 77.5°, and 81.7° correspond to (111), (200), (220), (311), and (222) planes of silver, respectively.^30^^,^^35^^,^^36^^,^^43^ The pattern is consistent with the standard Ag crystalline structure (JCPDF No. 04-0783) provided by the National Institute of Standards and Technology.^36^

**Figure 4:**
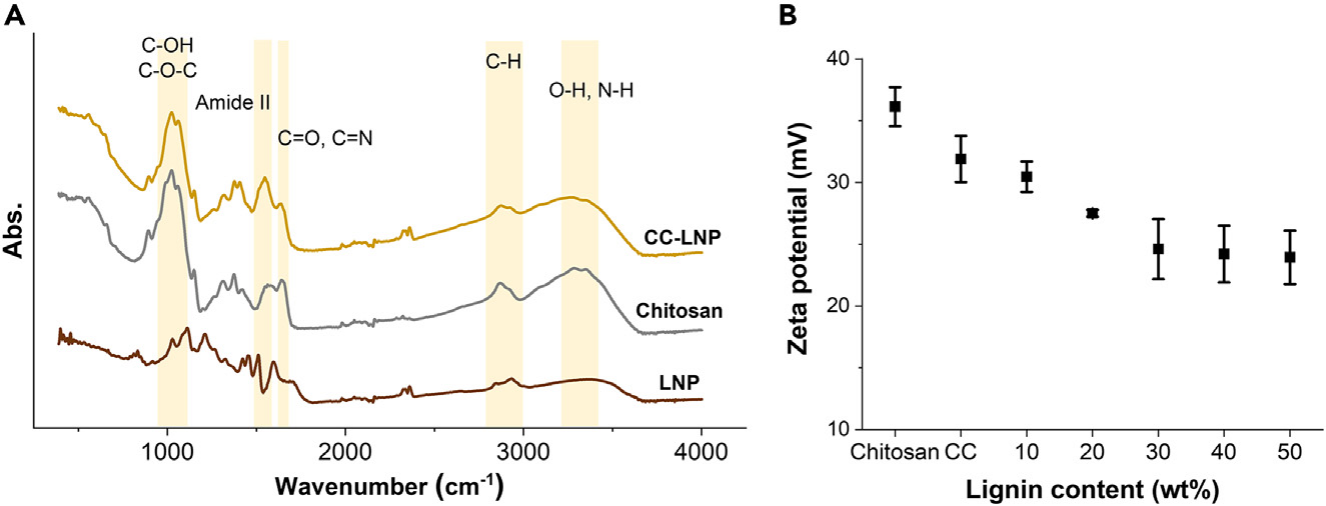
Interactions in the nanocomposite dispersion and films (A) FTIR spectra of LNP, chitosan, and CC-LNP. (B) Zeta (ζ) potential of the aqueous dispersion of chitosan, cross-linked chitosan (CC), and CC-LNP composites. Error bars represent standard deviation based on the entire population (n = 3).

The interactions of chitosan, glutaraldehyde, and LNPs in the composited suspension and films was revealed by recording the FTIR spectra and measuring zeta potential (Figure 4). Chitosan dissolves in water as a cationic polyelectrolyte at pH below its pKa 6.5.^44^ Owing to its primary amine groups, the zeta potential was + 37mV for chitosan solution at pH 4.5 (Figure 4B). When chitosan was cross-linked with glutaraldehyde, the zeta potential decreased, suggesting reactivity of the primary amines via imine chemistry. As shown in Figure 4A, the FTIR spectrum of CC-LNP showed a peak at 1,548 cm^−1^ (amide II^45^), which was absent from the spectrum of either chitosan or lignin. The interaction mechanisms between CC and LNPs are probably quite complex and involve electrostatic interactions at long-range and a series of short-distance forces such as hydrogen bonding and van der Waals forces. To further study the nature of interactions between LNP and CC, electrophoretic mobility measurements were carried out for different ratios of lignin/CC. The zeta potential decreased from +32 to +27 mV, when 20% LNP was added to CC (Figure 4B). This is likely because protonated primary amine groups of chitosan interacted with LNPs through double-layer and/or hydrogen bond interactions. It further decreased to +25 mV and remained at this plateau even with 50% LNP content, indicating that the LNPs were effectively covered by chitosan.

**Figure 5:**
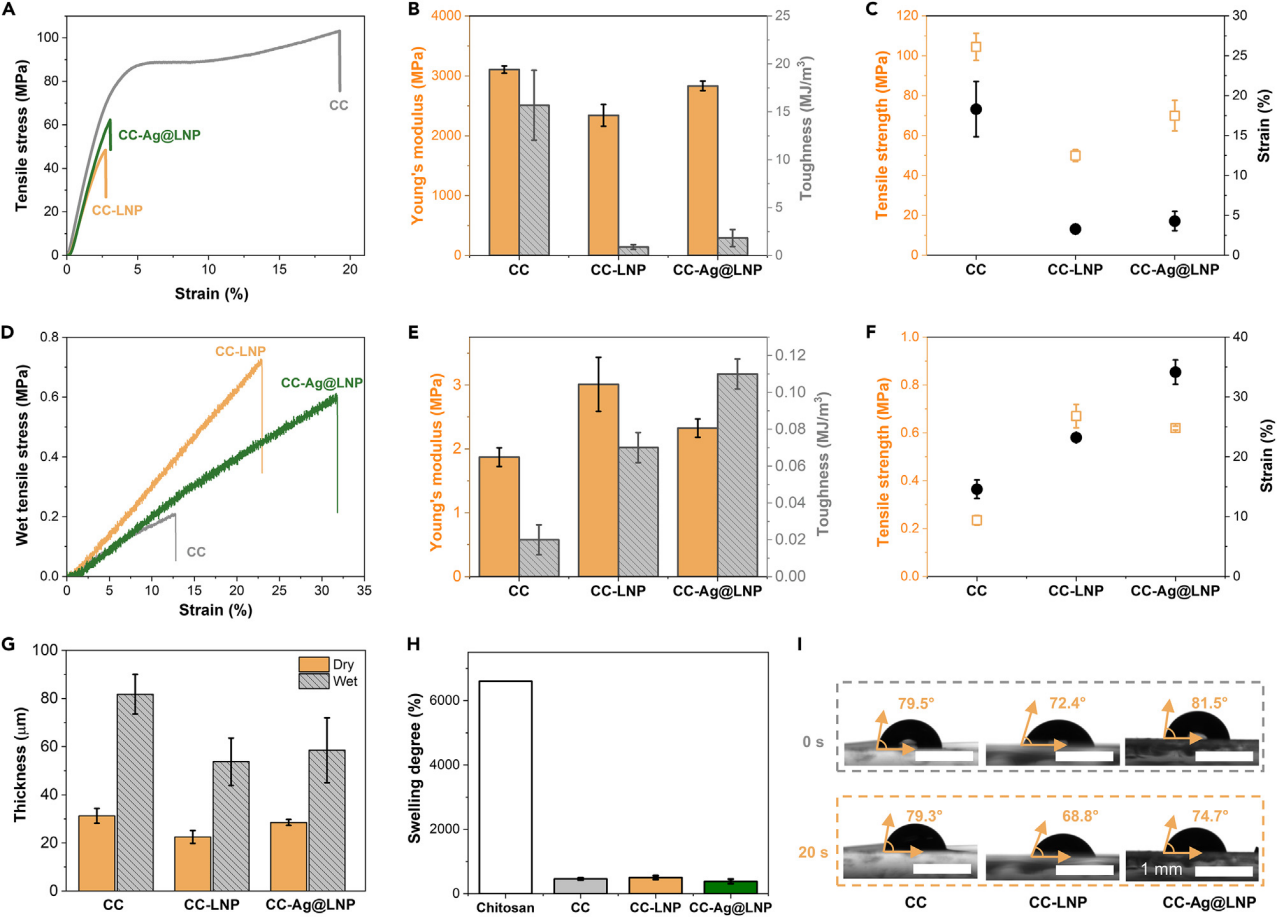
Mechanical properties and water stability of the nanocomposite films with a constant lignin content of 40 wt%. (A‒C) Mechanical properties of dry films. (D‒F) Mechanical properties of films immersed in water after 2 h. (G) Swelling degree of chitosan, CC, CC-LNP, and CC-Ag@LNP films immersed in water after 2 h. (H) Thickness of dry and wet CC, CC-LNP, and CC-Ag@LNP films. (I) Extraction of contact angle from obtained images of water drop on the given substrate (CC, CC-LNP, and CC-Ag@LNP films). Error bars represent standard deviation based on the entire population (n = 3). See also Figure S2.

The mechanical properties under wet condition are important when considering applications in contact with bacterial suspensions, but wet mechanical strength data are lacking for many antimicrobial films.^21^^,^^22^^,^^23^^,^^24^^,^^30^^,^^31^ Here, we studied the tensile strength of the chitosan-based films in dry and wet states. As shown in Figure 5A, the tensile strength of CC was 104.4 MPa and that of CC-LNP was 49.9 MPa, which is higher than other lignin-composited films.^29^^,^^30^ The tensile strength of CC-Ag@LNP (lignin content 40 wt%) was 69.9 MPa, which was higher than CC-LNP (lignin content 40 wt%). The Young’s modulus and toughness showed the same trend (Figure 5B). Nevertheless, the strains of CC-LNP and CC-Ag@LNP were very similar, 3.3% and 4.3%, respectively (Figure 5C). Interestingly, the films performed differently under wet condition. As shown in Figure 5D, the mechanical strength of CC-LNP (0.67 MPa) was three times that of CC (0.24 MPa) after immersing in water for 2 h. The tensile strength of CC-Ag@LNP was 0.62 MPa, which was only slightly lower than that of CC-LNP. Young’s modulus showed the same trend with tensile strength, that is, CC-LNP showed highest value of 3.0 MPa, CC was lowest (1.9 MPa), and CC-Ag@LNP (2.3 MPa) was in between the two. Under wet condition, LNPs and Ag@LNPs rendered the nanocomposite films (lignin content 40 wt%) stronger compared to the cross-linked chitosan alone, with toughness and strain at break increasing from CC (0.02 MJ/m^3^, 14.6%) to CC-LNP (0.07 MJ/m^3^, 23.2%) to CC-Ag@LNP (0.11 MJ/m^3^, 34.2%). Overall, the incorporation of LNPs in the cross-linked chitosan films resulted in lower dry strength, but improved wet strength, suggesting the possibility for use under moist conditions, for example, in contact with bacterial suspension.

We further investigated the stability of the films under wet condition by measuring the swelling degree and water contact angles. The swelling degree of CC, CC-LNP, and CC-Ag@LNP films were significantly lower than that of pure chitosan film (Figures 5G and 5H), which was consistent with the literature.^46^ Among the films, CC-Ag@LNP had the lowest swelling degree, which might explain its highest toughness (0.11 MJ/m^3^) and strain (34.2%). Comparing the thickness changes after immersion in water for 2 h, the changes of CC-LNP and CC-Ag@LNP films were notably smaller than that of CC, which might be the reason that their tensile strength and Young’s modulus were higher than that of CC alone. By comparing their wettability, we observed that CC-LNP showed lowest water contact angles both at the beginning of contact with water and after 20 s of contact (Figure 5I), implying that LNPs increased the hydrophilicity of the composited films. The hydrophilic nature of LNPs is well documented in the literature.^47^ The existence of LNPs decreased the swelling degree of chitosan-LNP composited films (Figure S2). When the lignin content increased from 0 to 100%, the swelling degree significantly decreased from 60 to 4 times of swelling relative to the original weight.

**Figure 6:**
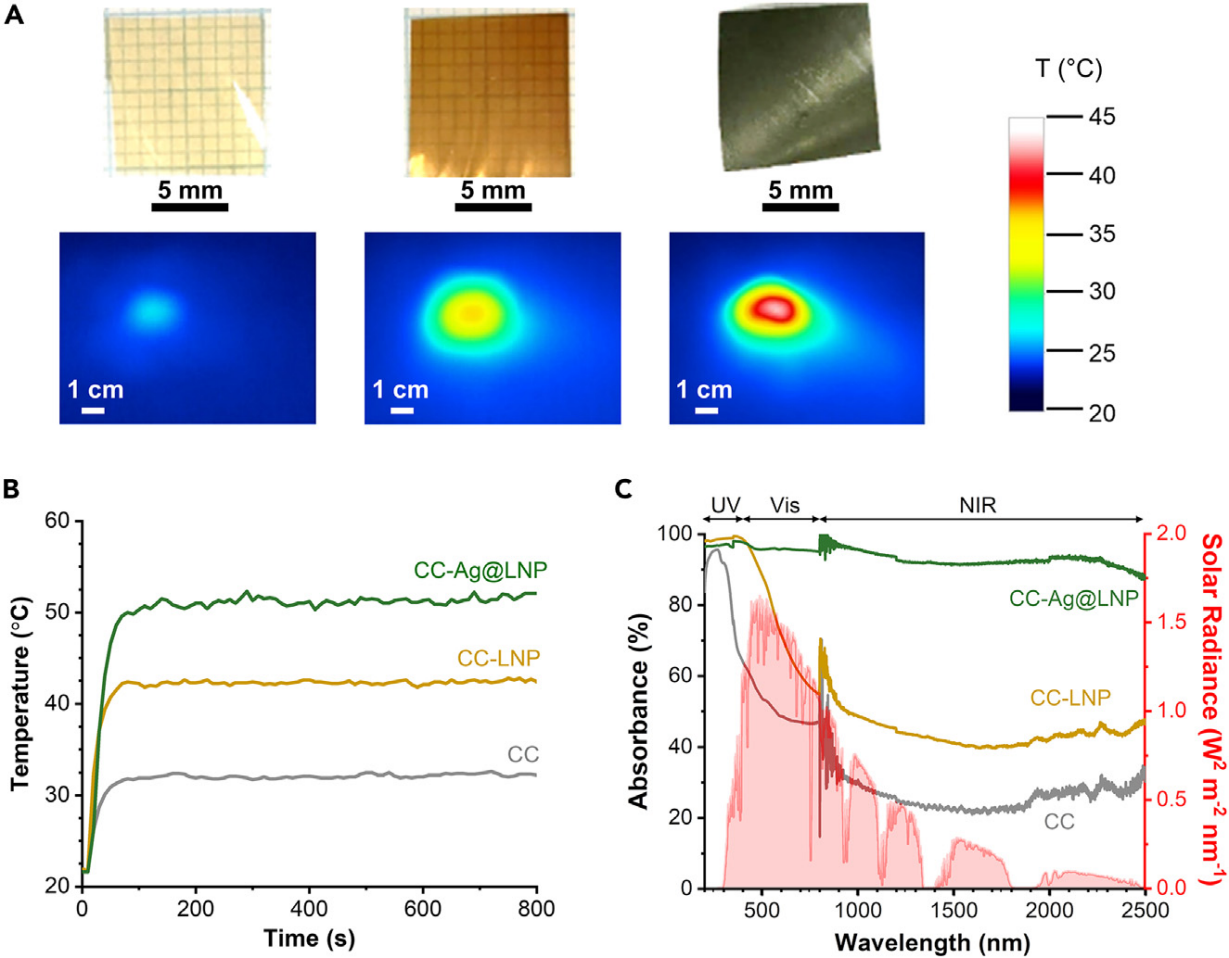
Photothermal and optical properties of the cross-linked chitosan and nanocomposite films with a constant lignin content of 40 wt% (A) Infrared images of irradiated CC (left), CC-LNP (middle), and CC-Ag@LNP (right) films after 15 min of exposure to simulated solar irradiation (0.1 W/cm^2^) with the corresponding digital images. Color map shows the temperature in °C. (B) Temperature changes of CC, CC-LNP, and CC-Ag@LNP films under artificial solar irradiation (0.1 W/cm^2^). (C) Absorption spectra of films calculated by A = 100% – T – R, where T = in-line (direct) transmittance (%) and R = reflectance spectra (%) of cross-linked chitosan (CC), CC-LNP, and CC-Ag@LNP films. The filled (red) spectrum indicates solar radiance at sea level. See also Figure S3.

To achieve stimuli-responsive triggered bactericidal properties, we first investigated the photothermal performance of the films. Infrared (IR) camera images demonstrated the photothermal properties of the films. After irradiation for 15 min by simulated solar light, CC-Ag@LNP showed highest temperature increment (Figure 6A). More accurate temperature changes during the irradiation were recorded by a temperature data logger (Figure 6B). All films reached a stable temperature after 110 s, with CC-Ag@LNP showing the highest equilibrium temperature of 52°C, followed by CC-LNP at 42°C, and CC at 31°C.

Absorption of light is crucial for photothermal heating. We measured the optical spectra of the three kinds of films to analyze their absorption (A) across the whole solar spectrum. We define here that A = 100% − T − R, where T is the transmittance and R is the reflectance of the films. The measured transmittance (T%) and reflectance (R%) spectra of the films are shown in Figure S3, and the calculated absorption (Abs.%) spectra are shown in Figure 6C. CC-Ag@LNP had the lowest transmittance and relatively low reflectance; therefore it absorbed 89% of the radiation across 200−2500 nm, i.e., the majority of the solar radiance, which is a priority requirement for high-efficiency photothermal materials.^48^ In the UV range (200–400 nm), CC-LNP provided the same absorption with CC-Ag@LNP, because their transmittance properties were equally low. In the visible range (400−800 nm), CC had the highest transmittance, CC-Ag@LNP was opaque, and CC-LNP was in between, which can also be predicted from their appearance (Figure 6A, digital photos). In the NIR range (800−2500 nm), both CC and CC-LNP exhibited similar transmittance values. However, CC-LNP demonstrated a lower reflectance compared to CC, resulting in a higher absorbance for CC-LNP than CC.

**Figure 7:**
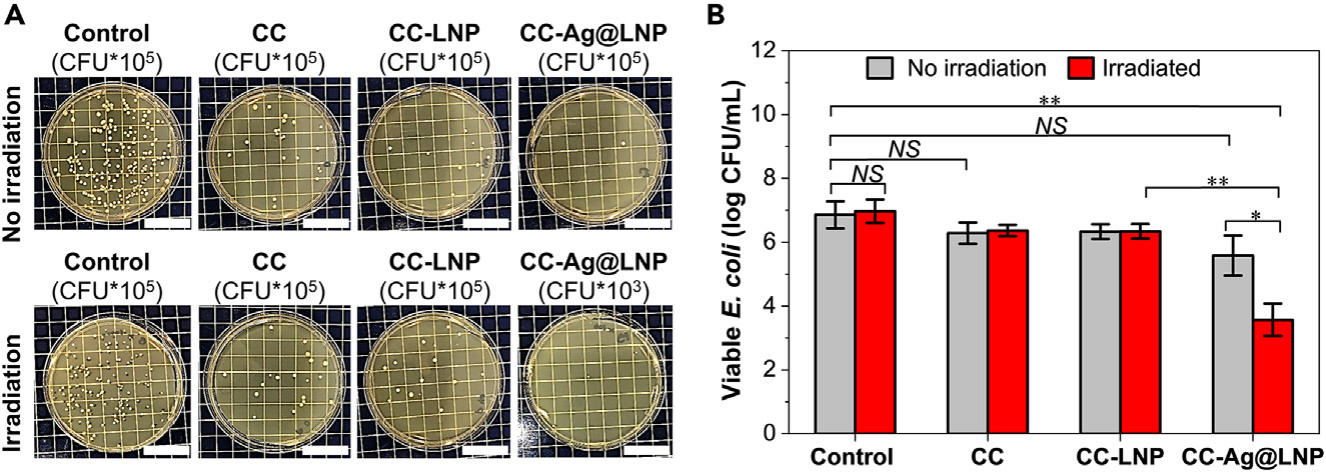
Antibacterial performance of the cross-linked chitosan and nanocomposite films with a constant lignin content of 40 wt% (A) Digital images of bacterial colonies of *E. coli* after different treatments; scale bar: 3 cm. (B) *E. coli* treated by CC, CC-LNP, and CC-Ag@LNP films for 15 min with or without simulated solar irradiation (0.1 W/cm^2^). Error bars represent standard deviation based on the entire population (n = 3). ∗p < 0.05 and ∗∗p < 0.01. NS (p > 0.05), statistically not significant. See also Figure S4, Tables S1 and S2.

After understanding the photothermal and optical properties of CC-Ag@LNP film, we tested the photothermal antibacterial properties of the prepared films using *E. coli* (for details, see experimental section and Figure S4). Survival of *E. coli* was followed and compared to the control suspension of *E. coli* suspended in water (Figure 7A). There was a slight reduction of similar magnitude in the colony forming units (CFU) due to contact with CC or CC-LNP films, but no significant difference, indicating that LNPs were not the active material. However, in the case of CC-Ag@LNP, the agar remained clean compared to the control samples, even when the dilution factor of the CC-Ag@LNP sample was 100-fold lower than that of the control sample. The survival of *E. coli* was calculated in CFU units and showed in Figure 7B. Analysis of variance was used to compare differences between two groups (Table S2). Clearly, for the control sample, the irradiation alone had no effect on the change of CFU, implying that the presence of the photothermal material is responsible for the effective sterilization. Table S1 summarizes the sterilization effect of different antibacterial materials which contain AgNP. It seems that materials with photothermal properties can kill bacteria effectively in a short time. In the present study, the CC-Ag@LNP with a low amount of silver (0.072 wt %) reduced cell viability by more than 99.9%. Such a good sterilization performance of CC-Ag@LNP may be stemming from the large surface area of AgNPs^6^ and superior broad-range absorbance of solar light as discussed previously.

## Discussion

We reported antibacterial films that can be activated photothermally by utilizing lignin nanoparticles and silver with cross-linked chitosan. The interactions between silver ions and lignin resulted in formation of finely dispersed AgNPs (∼3.3 nm) on the surfaces of colloidal lignin particles under mild reducing conditions (pH 3.8, room temperature). In addition to their superior antimicrobial effect against *E. coli* the composited films (CC-Ag@LNP) showed higher mechanical strength under wet condition compared to the cross-linked chitosan films. Such lignin-based nanocomposite films can be photothermally heated to 51°C in less than 2 min. The superior photothermal performance of the cross-linked chitosan film with silver nanoparticles residing on colloidal lignin particles originates from the efficient adsorption of radiation across the whole solar spectrum. Overall, this work expands the frontier of photothermally activated antibacterial films and elucidates the role of lignin as a multifunctional component in these sustainable materials.

### Limitations of the study

In our study, we synthesized bio-based nanocomposite films with enhanced wet strength and investigated their photothermal performance, as well as their potential for triggered antibacterial activity. However, the antioxidant activity of CC-Ag@LNP was lower than that of CC-LNP within 60 min (Figure S5). In addition, if these films are intended for use as freestanding materials under higher loads, additional improvements in wet strength may be necessary. With regards to the antibacterial activity, more bacteria could be tested side-by-side in future studies. Furthermore, the mechanism underlying photothermally activated sterilization remains unknown and warrants further investigation.

## STAR★Methods

### Key resources table

**Table:** 

REAGENT or RESOURCE	SOURCE	IDENTIFIER
**Chemicals, peptides, and recombinant proteins**		

Soda lignin	GreenValue LLC	PROTOBIND 2400
Silver nitrate	Sigma-Aldrich Sweden AB	CAS: 7761-88-8
Glutaraldehyde	Sigma-Aldrich Sweden AB	CAS: 111-30-8
Chitosan	Sigma-Aldrich Sweden AB	CAS: 9012-76-4

**Bacterial and virus strains**		

*Escherichia coli*	Sigma-Aldrich Sweden AB	Strain K12, lyophilized cells

**Software and algorithms**		

Origin	OriginLab	Origin 2021b
Chemdraw	ChemBioOffice	ChemDraw Professional 16.0
ImageJ	NIH	ImageJ 1.52a
DigitalMicrograph	Gatan	DigitalMicrograph
Quantax	Bruker	Quantax 70

### Resource availability

#### Lead contact

Further information and requests for resources should be directed to and will be fulfilled by the lead contact, Mika H. Sipponen (mika.sipponen@mmk.su.se).

#### Materials availability

This study did not generate new unique reagents.

### Method details

#### Preparation of lignin nanoparticles (LNPs) dispersion

LNPs were prepared by a solvent shifting method adapted from the literature.^29^ Specifically, 5 g of soda lignin was added into a solvent mixture composed of acetone: 300 g and deionized water: 100 g and stirred at 600 rpm for 3 h. Then the soda lignin solution was filtered to remove insoluble impurities. Deionized water (1200 mL) was then added to the soda lignin solution within 9 s. The size of LNPs was around 220 nm according to DLS result, the final concentration of the LNP dispersion was 0.27 wt%.

#### Preparation of LNPs supported silver nanoparticles (Ag@LNP) dispersion

Ag@LNP was prepared according to a UV irradiation method described in the literature.^31^ Specifically, 34 mg silver nitrate was dissolved in 2 mL of deionized water. Then the prepared silver nitrate solution (0.1 M) was added to 20 mL of aqueous dispersion of LNPs. Then the dispersion was irradiated under 366 nm UV lamp for 30 min.

#### Preparation of crosslinked chitosan film (CC), crosslinked chitosan-LNP film (CC-LNP) and crosslinked chitosan-Ag@LNP film (CC-Ag@LNP)

All films were prepared by a casting method. Petri dish (diameter: 3.5 cm) was used as a mould. Chitosan (1 g) was added to 100 mL deionized water which contains 0.572 mL acetic acid. For CC film, 2.95 g of prepared chitosan solution (1 wt%) and 180 μL of prepared glutaraldehyde solution (0.25 wt%) were mixed and poured into a petri dish. After evaporation under ambient temperature, the CC film was carefully removed from the mould. For CC-LNP film, the mixed solution contained 1.75 g chitosan solution, 180 μL glutaraldehyde solution and 3 mL LNP dispersion, with the other procedures were the same as above. For CC-Ag@LNP, Ag@LNP dispersion was used instead of LNP dispersion, and the other procedures were similar.

#### Dynamic light scattering and zeta potential measurement

A Zetasizer Nano ZS (Malvern, U.K.) was used to measure the size distribution and zeta potential of LNPs and chitosan-LNP composites. For particle size measurements by dynamic light scattering (DLS), 20 μL of LNPs dispersion was added to 2 mL DI water. The diluted LNPs dispersion samples were measured 3 times with 12 runs per run. Z-average size values based on intensity were reported. For zeta potential measurements by laser doppler electrophoresis, a dispersion sample was measured in a cuvette using a dip cell sensor, maximum voltage limited to 2 mV.

#### Electron microscopy

A JEM-2100 (JEOL Ltd., Japan) with a LaB_6_ filament was used to record the transmission electron microscopy (TEM) images of Ag@LNP with an accelerating voltage of 200 kV. The samples were prepared by depositing and drying dispersions on Cu grids and then loaded on a single tilt sample holder. A TM3000 (Hitachi, Japan) with a thermionic-type filament was used to record the scanning electron microscopy (SEM) images and energy-dispersive X-ray spectroscopy (EDS) spectrum of CC-Ag@LNP film with an accelerating voltage of 15 kV.

#### Fourier-transform infrared spectroscopy (FTIR)

A 670-IR spectrometer (Varian, U.S.A) was used to record the infrared (IR) spectrum of LNP and chitosan and their composites.

**X-ray diffraction (XRD)** A PANalytical X'Pert PRO MPD City (Malvern Instruments, UK) with Cu Kα radiation (λ = 1.5418 Å) was used to record the XRD pattern of the CC-Ag@LNP film.

#### UV-VIS-NIR spectroscopy

A Cary 5000 UV/Vis/NIR spectrometer (Agilent, U.S.A) was used to record the in-line (direct) transmittance and reflectance spectra of CC, CC-LNP, and CC-Ag@LNP films in the range of 200−2800 nm using air as background. The calculation of average spectral absorption (α) at range of 200−2500 nm was calculated according to the following equation^48^^,^^49^:

$$\alpha =\frac{\int_{\lambda 1}^{\lambda 2}A\left(\lambda \right)i\left(\lambda \right)d\left(\lambda \right)}{\int_{\lambda 1}^{\lambda 2}i\left(\lambda \right)d\left(\lambda \right)}$$

where A(***λ***) is spectral absorption, A = 100% − T − R (T and R are transmittance and reflectance, respectively). i(λ) is the solar spectral irradiance (W m^-2^ nm^-1^), obtained from ASTM standard G173-03.

#### Water contact angle measurements

A DSA25 Drop Shape Analyzer Contact Angle Meter (KRÜSS, Germany) was used to measure the wettability of films by means of the water contact angles by a sessile drop method.

#### Swelling degree measurement

A gravimetric method was used to calculate the swelling degree by measuring the weight of films before and after immersion in DI water for 2 h. The weight of wet samples were determined on an analytical balance after blotting the excess water with dry filter paper. The swelling degree was calculated by the following formula:

$$Swelling\;degree=\frac{\left(\text{Wt}-\text{Wd}\right)}{\text{Wd}}\times 100\%$$

where W_d_ is the initial dry mass and W_t_ is the mass of the wet sample.

#### Mechanical properties test

An Instron 5960 universal testing machine (Instron, U.S.A.) equipped with a 1 kN load cell was used to measure the mechanical properties of CC, CC-LNP, and CC-Ag@LNP films at a strain rate of 4 mm min^-1^. Films were cut to a rectangular shape with dimension of 25 × 3 mm. The mechanical properties in wet state were determined from films that had been immersed in DI water for 2 h immediately before the measurement. For each sample, three mechanical test experiments were repeated. The thicknesses of samples were measured by using an electronic outside micrometer (Schut Geometrical Metrology).

#### Photothermal performance testing

An ABA LED Solar simulator (Newport LSH-7320) with a light-emitting diode (LED) lamp was used to test the photothermal performance of CC, CC-LNP, and CC-Ag@LNP films under 1 sun radiation (0.1 W/cm^2^). A thermal camera (Testo 872) was used to record the infrared images of films after 15 min irradiation. A temperature data logger (Testo 175 T3) was used to record the temperature change during the irradiation.

#### **Bacterial s**urvival test

The antibacterial activity of CC, CC-LNP, and CC-Ag@LNP films was tested by using a plate counting method. Firstly, frozen *E. coli* was added in DI water to prepare *E. coli* suspensions (OD_600_ ≈ 0.5). Then films were punched into circles of 6 mm in diameter and immersed in *E. coli* suspensions (OD_600_ ≈ 0.5) in a 96-well plate for 15 min with/without irradiation. After the treatment, the bacterial suspensions were diluted 10 – 100000-fold. 100 μL of the diluted suspension was spread on a LB agar culture media plate. The agar plates were incubated at 37 °C for 12∼ 24 h, and the antibacterial activity was evaluated by counting the colony-forming units (CFU) on the agar plates. The extent of survival was determined relative to the number of CFU/mL in the bacterial suspension which were treated with films. For example, the countable plate had 127 colonies with a dilution factor of 10000, then $\log\left(\frac{CFU}{mL}\right)=\log\left(\frac{127\times 10000}{0.1}\right)=7.1$.

#### Antioxidant activity test

A freshly prepared ABTS·^+^ radical solution was diluted (1 : 100) until it reached an absorbance of 0.6 at 734 nm. Then, a piece of CC-LNP or CC-Ag@LNP film (30 × 90 mm) were immersed in 3 mL of ABTS·^+^ radical solution and shaken at 25 °C for 1 h. The antioxidant radical scavenger activity was calculated according to the reduction of absorbance of the ABTS·^+^ radical at 734 nm.

## Data Availability

All data used in this paper is available in the main text, in the supplementary information, or the sources have been clearly stated. Any additional information required to reanalyze the data reported in this work is available from the lead contact upon reasonable request.

## Supplemental information

Document S1. Figures S1‒S5 and Tables S1 and S2
