# Supplementary material for: Ag-lignin hybrid nanoparticles for high-performance solar absorption in photothermal antibacterial chitosan films
Source: iScience. 2023 Sep 25;26(10):108058. doi: 10.1016/j.isci.2023.108058 (PMC10579425; doi:10.1016/j.isci.2023.108058)
Supplement: Document S1. Figures S1‒S5 and Tables S1 and S2 [file mmc1.pdf]

iScience, Volume *26*

Supplemental information

Ag-lignin hybrid nanoparticles for high-performance solar absorption in photothermal antibacterial chitosan films

Jinrong Liu and Mika H. Sipponen

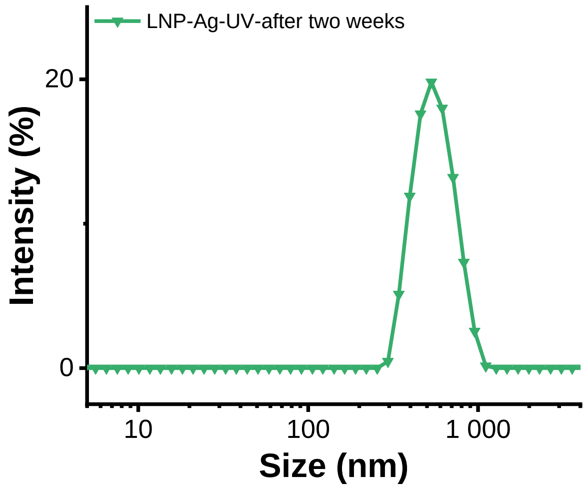

Figure. S1 Diameter distribution of LNP-Ag-UV-after two weeks from DLS measurement. Related to Figure 2.

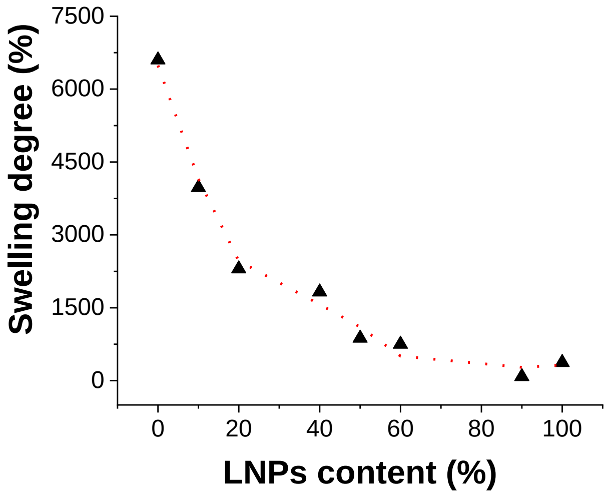

Figure. S2 Swelling degree (%) of chitosan-LNP composites. Related to Figure 5.

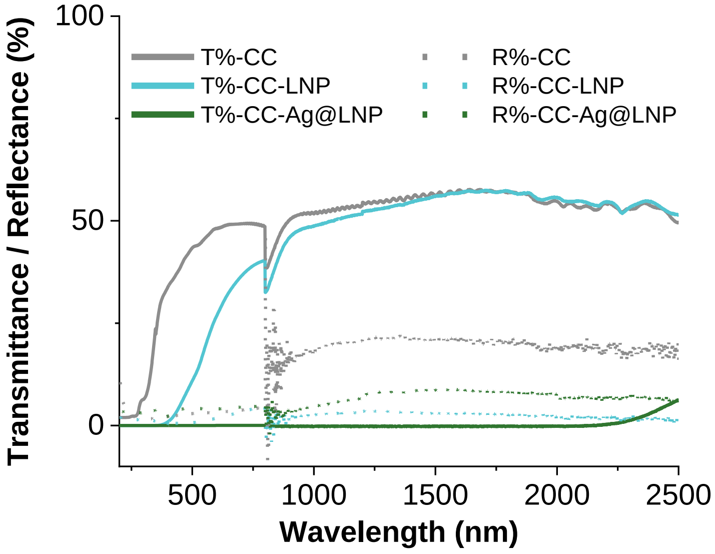

Figure. S3 Transmittance and reflectance spectra of crosslinked chitosan (CC), CC-LNP and CC-Ag@LNP films, where T = in-line (direct) transmittance (%) and R = reflectance spectra (%). Related to Figure 6.

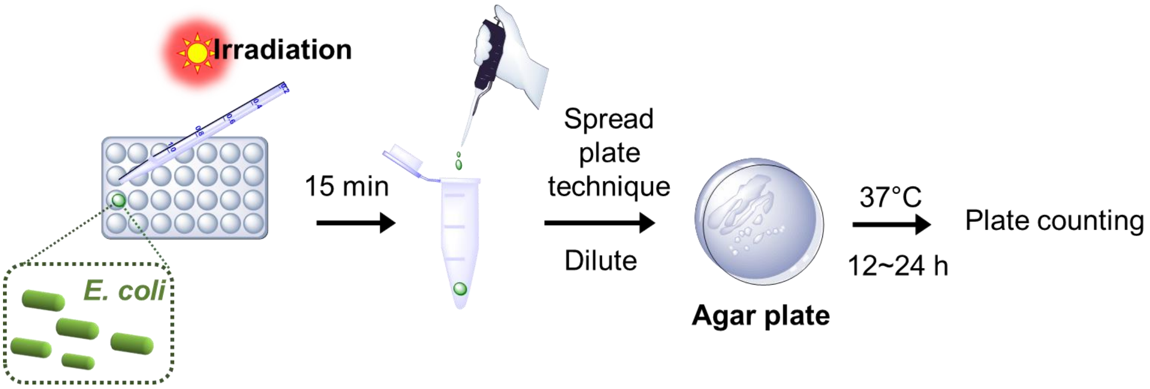

Figure. S4 Scheme of testing photothermal antibacterial properties. Related to Figure 7.

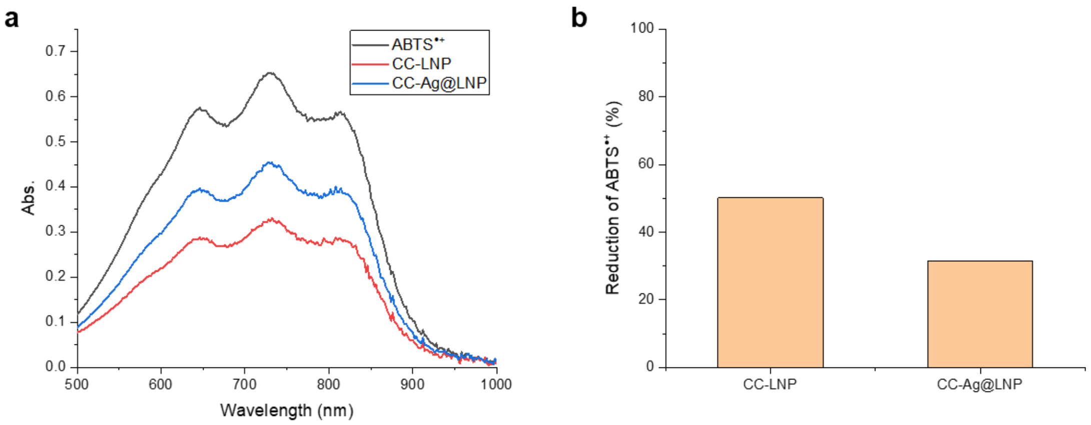

Figure. S5 (a) Absorbance spectra of $ABTS^{•+}$ solution and $ABTS^{•+}$ solution with CC-LNP or CC-Ag@LNP. (b) Antioxidant properties of CC-LNP and CC-Ag@LNP films. CC-LNP and CC-Ag@LNP were immersed in $ABTS^{•+}$ solution at 25 °C after 1 h. Related to STAR Methods.

Table S1. The method of preparing AgNP and the bactericidal performance of various antibacterial materials. Related to Figure 2 and 7.

**Antibacterial material**	**Reducing agent**	**Adjust pH to alkaline**	**Average AgNP size (nm)**	**Bacterial strains**	**Irradiation**	**Time (min)**	**Sterilization effect**	**Ref**
Dispersion (Ag@rGO-Fe_3O_4-PEI)	ascorbic acid	need	> 10 nm	*E. coli*	Yes	20	99.9%	[S1]
Hydrogel (10GGMMA/ 0.1LNP@Ag)	LNP	need	8 – 21 nm	*E. coli*, *S. aureus*	No	60	28% 31%	[S2]
Hydrogel (10GGMMA/ 0.5LNP@Ag)	LNP	need	8 – 21 nm	*E. coli*, *S. aureus*	No	60	91% 78%	[S2]
Film Type III	chitosan	no need	< 10 nm	*E. coli*	No	150	> 99%	[S3]
Film (AgNPs@EL-BC1.85)	ethanol-extracted lignin	need	> 10 nm	*E. coli*, *S. aureus*	Yes	10	88%, 68%	[S4]
Film (AgNPs@EL-BC6.09)	ethanol-extracted lignin	need	> 10 nm	*E. coli*, *S. aureus*	Yes	10	> 99.99%, > 99%	[S4]
Film (WPU+LS−PDA−Ag-5%)	sodium lignosulfonate	need	> 10 nm	*E. coli*	Yes	10	> 99.9%	[S5]
Film (CC-Ag@LNP)	LNP	no need	< 10 nm	*E. coli*	Yes	15	> 99.9%	This work

Table S2. Analysis of variance of two groups. Related to Figure 7.

**Samples**	***P-value***	
Control_No & Control_Ir	0.886225	*NS*
Control_No & CC_No	0.206386	*NS*
Control_No & CC-Ag@LNP_No	0.074694	*NS*
Control_No & CC-Ag@LNP_Ir	0.002156	**
CC-LNP_Ir & CC-Ag@LNP_Ir	0.002148	**
CC-Ag@LNP_Ir & CC-Ag@LNP_Ir	0.023809	*

The significance level is usually set at 0.05. *P < 0.05 and **P < 0.01. NS (P > 0.05), not statistically significant.

Supplemental References

[S1] N. Wang, B. Hu, M. L. Chen and J. H. Wang, Nanotechnology, 2015, 26, 195703.

[S2] L. Wang, Q. Wang, A. Slita, O. Backman, Z. Gounani, E. Rosqvist, J. Peltonen, S. Willför, C. Xu, J. M. Rosenholm and X. Wang, Green Chem., 2022, 24, 2129–2145.

[S3] D. Wei, W. Sun, W. Qian, Y. Ye and X. Ma, Carbohydr. Res., 2009, 344, 2375–2382.

[S4] Y. Zhang, D. Yang, X. Qiu and Z. Li, ACS Appl. Bio Mater., 2022, 5, 5943–5952.

[S5] Y. Zhang, Y. Wang, Z. Li, D. Yang and X. Qiu, ACS Appl. Bio Mater., 2022, 5, 4256–4263.
